# Notes and News

**Published:** 1894-04-07

**Authors:** 


### Notes and News.

Collected and Communicated.

MEETINGS OF THE WEEK.

London Homœopathic Hospital, 35, Queen's Square, April 10th, 3·30.

Miss Emily W. Dixon, of Dublin, has been elected the Female fellow of the Royal College of Surgeons, Ireland.

The sad fate of a druggist's boy shows that familiarity with medicine is not free from the contempt which is proverbially associated with that state. An incautious youth made use of his Easter holiday to indulge in thirty oranges, a cocoa-nut, a mince pie, cider, mineral water champagne, tea, cake, and lemonade in one day. This immoderate diet resulted in his death.

A new hospital for male tuberculous children is to be opened at Villiers-sur-Marne, France. It is intended to accommodate eighty patients. The heating apparatus and ventilating and other arrangements will occupy the basement. Above this are the wards, nearly 40 feet high, around which run galleries in which are the beds, leaving a central space. The cubic space will be 120 cubic metres per bed. A very complicated system of ventilation is to be adopted, and medicated air is to be supplied to the wards both morning and evening. A similar hospital for female children exists at Villepinte.

A meeting of the Council of the London and Counties Medical Protection Society was held at 57, Harley Street, on Tuesday, March 20th. Twenty-four new members were elected, and Mr. Howard Marsh was nominated as a vice-president of the Society. A deputation on behalf of Dr. Anderson was introduced by the Earl of Stamford, and resolutions were passed condemnatory of the treatment Dr. Anderson had received, and in favour of supporting his appeal to the Privy Council. It was decided that the annual dinner of the Society should be held at the Restaurant Frascati in Oxford Street on Tuesday, June 19th, and that ladies should be invited to be present.

Mr. Rider Haggard and Mr. Jerome K. Jerome are going to read selections from their works at a drawing-room meeting, in aid of the North-Eastern Hospital for Children, Hackney Road, to take place at 84, Harley Street, on April 11th, when Canon Barker will plead the cause of the hospital, which is in great straits for money.

The annual reports of the larger hospitals, for the year ending December, 1893, are now coming to hand, and it is satisfactory to note that the more important provincial hospitals, and some of the best managed smaller and cottage hospitals, have adopted the uniform system of accounts. The General Hospital, Birmingham, the Royal Infirmary, Newcastle, and the Cumberland Infirmary, reports of which are just to hand, have all adopted this system. We shall hope to find that the example thus set will be universally followed, in which case the advantage to the institutions and their supporters will undoubtedly be great. We hope that many secretaries and committees may see their way to give up one page of the report in order to call the attention of their subscribers to the contents of "The Hospital Annual," a book which cannot fail to be of great interest, whilst its perusal must tend to loosen the purse-strings and increase the intelligent interest taken in the institutions by the inhabitants of the district which each hospital serves.

The admirable arrangements for cancer patients at the Middlesex Hospital have proved so beneficial that an effort is being made by the board to increase the accommodation. The cancer wards of this institution are unique in their way. In the year 1792 Mr. Samuel Whitbread fitted up a ward for the admission of patients afflicted with cancer, and settled the interest of £4,000 Three per Cent. Consolidated Bank Annuities for ever as an endowment in aid of the cancer establishment of the hospital. Since then the cancer fund has been augmented by other donations and bequests. These endowments enabled the governors to appropriate three wards exclusively to females suffering from cancer, besides providing accommodation for men afflicted with the same disease. A wise provision made by the founders of the cancer establishment was to the effect that the refuge should be permanent. To this end it was directed that the patients might remain until "relieved by art or released by death." At present there are 35 cancer beds—nine for men and 26 for women. These are always occupied. It is an unfortunate fact that cancer is on the increase, and there are now on the books of the hospital 35 applicants waiting to come in. In the course of last year the governors obtained possession of a valuable site in Nassau Street, adjacent to the hospital, upon which it is proposed to erect new accommodation for female cancer patients. The hospital would thus be relieved of a class of case unsuited to the ordinary wards, and the present female cancer wards be rendered available for the general purposes of the hospital. The Prince of Wales has shown great interest in the scheme, which will cost about £12,000 to carry out, and the weekly board hope that at the festival dinner on April 13th, his Royal Highness, who will take the chair on the occasion, will be able to announce that this highly-desirable addition to the hospital will be opened free of debt.

